# A polypill strategy for lipid lowering and anti-platelet therapy after acute coronary syndrome: A pilot randomized controlled trial

**DOI:** 10.1016/j.ajpc.2026.101499

**Published:** 2026-03-07

**Authors:** Juan David Coellar, Neil Keshvani, Anand K. Jain, Muhammad Shariq Usman, Matthew W. Segar, James W. Miller, Myriam Bustillo-Rubio, Dharam J. Kumbhani, Mohanakrishnan Sathyamoorthy, Javed Butler, Subhash Banerjee, Sean Yates, Thomas J. Wang, Ambarish Pandey

**Affiliations:** a Division of Cardiology, Department of Medicine, UT Southwestern Medical Center, Dallas, TX, USA; b Baylor Scott and White Research Institute, Dallas, TX, USA; c Baylor Scott and White The Heart Hospital Plano, Plano, TX, USA; d Division of Cardiology, Department of Medicine, Texas Heart Institute, Houston, TX, USA; e Department of Internal Medicine, Burnett School of Medicine at Texas Christian University, Fort Worth, TX, USA; f Department of Medicine, University of Mississippi, Jackson, MS, USA; g Baylor Scott and White Heart and Vascular Hospital, Dallas, TX, USA; h Department of Cardiology at Baylor University Medical Center, Dallas, TX, USA; i Department of Pathology, UT Southwestern Medical Center, Dallas, TX, USA; j University of Michigan School of Medicine, Ann Arbor, Michigan, USA

**Keywords:** Acute coronary syndrome, Polypill, Lipid, Platelet aggregation, Adherence

## Abstract

**Background:**

Medication non-adherence following acute coronary syndrome (ACS) is common, especially among underserved populations. The use of fixed-dose combination pills (polypills) may improve both adherence and prescription practices.

**Methods:**

This open-label, two-center pilot RCT enrolled adults within 30 days of ACS with stent placement and randomized them 1:1 to a once-daily polypill (aspirin 81 mg, rosuvastatin 40 mg, and clopidogrel 75 mg or prasugrel 10 mg) versus usual care. Key endpoints were follow-up LDL-C levels and platelet reactivity, measured by impedance aggregometry (ohms [Ω]; lower=worse aggregation/better inhibition) at 30 days. Medication adherence was assessed with the Morisky Medication Adherence Scale (range 0–8, higher=better adherence). Treatment effects were reported as least squares means (LSM) differences using baseline-adjusted linear regression models.

**Results:**

Among 140 randomized participants (median age 58 years, 29 % female, 14 % Black, 63 % Hispanic), 128 (91 %) completed follow-up. Index ACS events were STEMI (29 %), NSTEMI (51 %), and unstable angina (19 %). There was no significant difference between polypill and usual care in LDL-C (LSM difference: -4.58 mg/dL, 95 % CI -12.68 to 3.52, *p* = 0.27) and platelet reactivity (-0.08Ω, 95 % CI -0.96, 1.11, *p* = 0.88) at 30 days. Similarly, medication adherence was similar between groups at 30 days (MMAS-8 LSM difference: 0.13, 95 % CI -0.23 to 0.49, *p* = 0.47). Composite emergency department visits and hospitalizations over 30 days were similar between groups (polypill vs usual care: 0.14 vs 0.18 events/participant, *p* = 0.67).

**Conclusions:**

This pilot RCT demonstrated the feasibility of a polypill strategy incorporating P2Y12 inhibitors, aspirin, and high-intensity statin in the post-ACS period. Larger, longer-duration trials are needed to evaluate clinical effects.

**Registration:** NCT05514938

## 1 Introduction

Acute coronary syndromes (ACS) represent a major public health challenge, affecting approximately 1 million Americans annually and contributing substantially to cardiovascular morbidity and mortality [1]. Despite significant advances in management, patients remain at heightened risk for adverse cardiovascular events in the immediate post-ACS period, with annualized rates of major adverse cardiovascular events reaching 41 % within the first 30 days compared to 6.4 % at ≥1 year [2]. Current multi-society guidelines recommend dual antiplatelet therapy (DAPT) consisting of aspirin and a P2Y12 inhibitor for 12 months, combined with high-intensity statin therapy [3]. These pharmacotherapies have demonstrated substantial reductions in recurrent ischemic events, stent thrombosis, and cardiovascular mortality when used consistently in the post-ACS period [4].

Despite the proven efficacy of evidence-based therapies, medication non-adherence represents a critical barrier to optimal outcomes in the post-ACS period. Up to one-third of patients discontinue P2Y12 inhibitors and high-intensity statins within a few months of the ACS event, and the premature discontinuation of these therapies is associated with substantially higher risk of death and in-stent thrombosis [[5], [6], [7], [8]]. Moreover, non-adherence rates are particularly high among racial/ethnic minorities and socioeconomically disadvantaged patients [6,7,[9], [10], [11], [12]]. Financial barriers, complex medication regimens, and limited healthcare access contribute to these disparities, highlighting the need for innovative implementation strategies [9].

The polypill approach, which combines multiple evidence-based medications into a single daily formulation, has emerged as a promising strategy to improve medication adherence and clinical outcomes [13]. Polypill interventions significantly improve medication adherence and reduce cardiovascular risk factors in both primary and secondary prevention settings [14]. Furthermore, the SECURE trial demonstrated polypill effectiveness in secondary prevention, demonstrating a 24 % relative risk reduction in cardiovascular death, non-fatal myocardial infarction, or urgent coronary revascularization among older patients age ≥ 75 years with recent MI within the previous 6 months treated with a combination of aspirin, ramipril, and atorvastatin [15]. However, little data exist for the feasibility and safety of a polypill in the immediate post-MI vulnerable period when the risk associated with non-adherence to therapies such as P2Y12 inhibitors is the highest [6]. Furthermore, the feasibility of a polypill strategy among underserved patients with ACS, who are at the highest risk of non-adherence, is not known. Accordingly, we conducted a pilot, implementation-focused RCT to evaluate the feasibility, safety, and potential treatment effects of a polypill strategy on biological surrogate measures of adherence to lipid lowering and anti-platelet therapies.

## 2 Methods

### 2.1 Study design and setting

We conducted a two-center, open-label randomized controlled trial comparing a polypill strategy versus usual care in adults in the immediate post-ACS period. The study was conducted at Parkland Hospital, an urban, county safety-net hospital, and Clements University Hospital, an academic hospital at UT Southwestern Medical Center, in Dallas, Texas. These sites serve racially diverse populations including both insured and uninsured patients within Dallas County. The trial was approved by the Institutional Review Board at UT Southwestern Medical Center, and all participants provided written informed consent before enrollment. The trial was registered at ClinicalTrials.gov (NCT05514938).

English and Spanish speaking adults aged 18 years and older were eligible for enrollment if they had been hospitalized for ACS, underwent percutaneous coronary intervention with drug-eluting stent placement, and were discharged on DAPT with prasugrel or clopidogrel and statin therapy. Potential participants were identified through electronic health record screening and contacted both during and after hospital discharge to assess eligibility and interest in study participation. Participants were eligible for the study if able to be randomized within 30 days of hospital discharge from their index ACS event and prescribed a statin, aspirin, and either clopidogrel or prasugrel on discharge. Key exclusion criteria included severe kidney dysfunction (estimated glomerular filtration rate <30 mL/min/1.73m²), severe liver dysfunction, known allergy to study medications, pregnancy, and inability to provide informed consent (**Supplemental Table 1**).

### 2.2 Randomization and interventions

Participants were randomized in a 1:1 ratio to either polypill strategy or usual care using a computerized randomization algorithm (**Central Illustration**). The randomization sequence was maintained by a senior pharmacist with no involvement in the trial. The polypill contained aspirin 81 mg, rosuvastatin 40 mg, and either clopidogrel 75 mg or prasugrel 10 mg, formulated for once-daily dosing. Two formulations were available based on the attending physician's choice of P2Y12 inhibitor at discharge. Ticagrelor was not included in the polypill given its twice daily dosing. The polypill was supplied as an inert gel capsule containing each component medication without chemical combination or compounding. Participants randomized to the polypill arm received a 30-day supply of study medication at the baseline visit, provided directly by the research team, with instructions to take one capsule daily for the entire 30-day study period. Participants in the usual care arm continued the same individual medications prescribed separately at time of discharge according to standard clinical practice. All copayments and medication costs during the study period were covered to eliminate financial barriers between treatment groups. All participants received standard-of-care follow-up with interventional cardiology within 30 days of hospital discharge, allowing for medication refills and reconciliation as needed. Discharge prescriptions ranged from 30–90 days as determined by the discharging clinical team.

### 2.3 Study visits and assessments

The combined baseline, screening, and randomization visit occurred after hospital discharge from the index ACS event, during which informed consent was obtained, medical and social history were recorded, and vital signs were measured. Baseline non-fasting blood samples were collected for comprehensive lipid panels and platelet reactivity testing, and participants completed baseline questionnaires assessing medication adherence, treatment satisfaction, and quality of life. The follow-up visit occurred at 30 days post-randomization, during which participants returned for repeat laboratory testing (lipid panel, platelet reactivity), vital sign assessment, and completion of follow-up questionnaires. At the conclusion of this visit, participants in the polypill arm were transitioned back to individual component medications to continue their standard post-ACS care, coordinated with their outpatient providers.

Comprehensive lipid panels were analyzed using the Abbott Alinity-c chemistry analyzer platform with standardized laboratory protocols and quality control procedures. Low-density lipoprotein cholesterol (LDL-C) concentrations were calculated using the Friedewald equation based on measured total cholesterol, high-density lipoprotein cholesterol, and triglyceride levels [16]. For participants with triglyceride levels exceeding 400 mg/dL, direct LDL-C measurement was performed using enzymatic methodology to ensure accuracy, as the Friedewald equation becomes unreliable at elevated triglyceride concentrations. Platelet aggregation response assessment utilized whole blood impedance aggregometry performed with the Chrono-log Model 592 Whole Blood Aggregometer following standardized laboratory protocols [17]. Whole blood samples were collected in sodium citrate anticoagulation tubes and processed within two hours of collection to maintain specimen integrity. Samples were diluted 1:1 with 0.9 % normal saline solution and incubated at precisely 37 °C for five minutes before testing. Adenosine diphosphate agonist was added at concentrations of 10 μM to stimulate platelet aggregation response, with results recorded as maximum impedance change measured in ohms (Ω). All laboratory analyses were performed by certified clinical laboratory scientists following Clinical Laboratory Improvement Amendments standards and institutional quality assurance protocols, with platelet reactivity testing performed by technicians blinded to treatment group assignment. Medication adherence was evaluated using the 8-item Morisky Medication Adherence Scale (MMAS-8) [18], treatment satisfaction using the Treatment Satisfaction Questionnaire for Medication Version 1.4 (TSQM) [19,20], and quality of life using the Seattle Angina Questionnaire (SAQ) [21].

### 2.4 Endpoints

Key study endpoints were LDL-C and platelet aggregation response at 30 days. Platelet aggregation response values ranged from 0–16 Ω. Lower impedance values indicate superior platelet inhibition, with values of ≤4 Ω considered to represent an adequate therapeutic P2Y12 antiplatelet effect based on established laboratory standards [17]. Secondary endpoints included medication adherence (MMAS-8), treatment satisfaction (TSQM), and quality of life (SAQ) at 30 days. The MMAS-8 scores medication-taking behaviors from 0–8, with higher scores indicating better adherence (scores <6 indicate low adherence, 6 to <8 medium adherence, and 8 high adherence) [18]. The TSQM and SAQ are scored 0–100, with higher scores representing greater satisfaction and better health status, respectively. Exploratory clinical outcomes included emergency department (ED) visits and all-cause hospitalizations over the 30-day follow-up period.

### 2.5 Statistical analysis

Baseline characteristics were compared between treatment groups using chi-square or Fisher's exact tests for categorical variables and two-sample *t*-tests or Wilcoxon rank-sum tests for continuous variables, as appropriate. Continuous variables are presented as median [interquartile range] and categorical variables as frequencies and percentages. Key outcomes were assessed with both within-group and between-group analyses.

Within-group changes from baseline to 30 days were assessed using paired *t*-tests for each treatment arm separately, with results presented as mean change with 95 % confidence intervals. Between-group comparisons at 30 days were performed using least squares means (LSM) analysis from linear regression models. All between-group models were adjusted for baseline values of the respective outcome measure and treatment group assignment. Similarly, patient-centered endpoints including MMAS-8, TSQM, and SAQ were analyzed using both within-group paired *t*-tests and between-group least squares mean comparisons, with models adjusted for baseline values of the respective outcomes. We conducted an exploratory subgroup analysis among participants with baseline medication adherence scores <6 on the MMAS-8 to examine treatment effects in patients with poor baseline adherence.

Total ED visits and hospitalizations were assessed over the 30-day study period, with composite events calculated as the sum of all ED visits and hospitalizations divided by the number of participants in each treatment group. Between-group comparisons of clinical outcomes were performed using negative binomial regression models, with results presented as events per participant over the 30-day follow-up period and corresponding p-values. Available case analysis was performed, including only participants who completed both baseline and 30-day assessments. Statistical significance was defined as *p* < 0.05 for all outcomes. Analyses were performed using R version 4.4.2 (R Foundation For Statistical Computing, Vienna, Austria).

## 3 Results

**Fig. 1:**
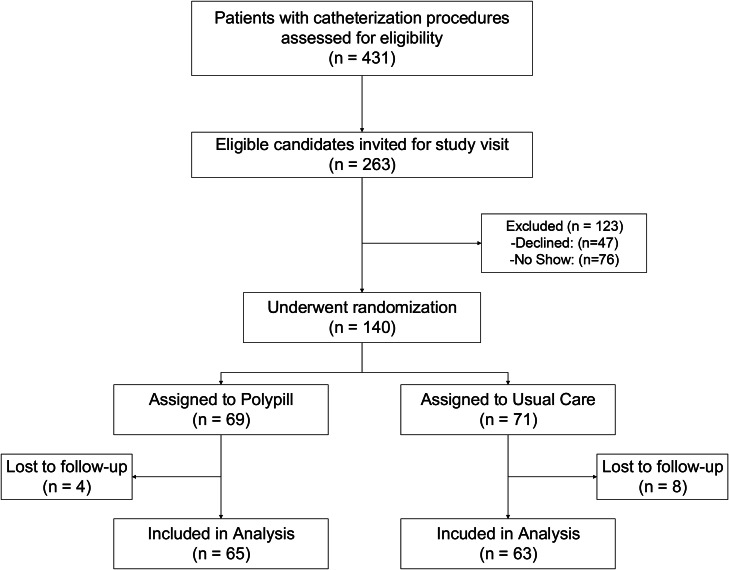
CONSORT Diagram Caption: Flow diagram showing participant enrollment and randomization.

Between December 2022 and April 2025, we reviewed 431 patients with left heart catheterization procedures for eligibility and invited 263 candidates for baseline study visit. A total of 140 patients provided informed consent and were randomized to polypill (*n* = 69) or usual care (*n* = 71, Fig. 1). Twelve participants (*n* = 4 in polypill group, *n* = 8 in usual care) were lost to follow-up during the 30-day study period, resulting in 128 participants (91.4 %) who completed the study protocol and were included in the primary analysis.

Participants were enrolled a median of 15 days (IQR 10, 22) following their initial percutaneous coronary intervention. Among the 140 participants randomized, the median age was 58 years, with 71 % being male (Table 1). The study population was racially diverse, with 63 % of self-reported Hispanic ethnicity and 14 % of Black race, and 46 % identified Spanish as their primary language. The cohort represented a predominantly socioeconomically disadvantaged population, with 86 % receiving care through the safety-net hospital system and 63 % either uninsured or relying on county indigent care support. Cardiovascular risk factors were prevalent, with more than half having diabetes mellitus (56 %), the vast majority having hypertension (85 %) and hyperlipidemia (88 %), and a high proportion with current or past tobacco use (43 %).

**Table 1:** Baseline characteristics of the study cohort.

Characteristic	Overall (*N* = 140)	Usual Care (*N* = 71)	Polypill (*N* = 69)	P-value
Age, years	58 (51, 64)	61 (53, 67)	55 (50, 62)	0.007
Male sex	99 (71)	51 (72)	48 (70)	0.8
Race/Ethnicity				0.3
White	114 (81)	59 (83)	55 (80)	
Black or African American	20 (14)	8 (11)	12 (17)	
Asian	5 (3.6)	4 (5.6)	1 (1.4)	
Other	1 (0.7)	0 (0)	1 (1.4)	
Hispanic ethnicity	88 (63)	47 (66)	41 (59)	0.4
Primary language Spanish	65 (46)	36 (51)	29 (42)	0.3
Receives care at county hospital	121 (86)	59 (83)	62 (90)	0.2
Insurance Status				0.5
Private health insurance	30 (21)	14 (20)	16 (23)	
Medicare	16 (11)	10 (14)	6 (8.7)	
Medicaid	4 (2.9)	2 (2.8)	2 (2.9)	
County Indigent Support	51 (36)	22 (31)	29 (42)	
Uninsured	38 (27)	22 (31)	16 (23)	
Other public source	1 (0.7)	1 (1.4)	0 (0)	
Education Status				0.7
Less than high school	42 (30)	23 (32)	19 (28)	
High school degree or GED	30 (22)	15 (21)	15 (22)	
Some college without degree	21 (15)	9 (13)	12 (18)	
Associate's degree	5 (3.6)	2 (2.8)	3 (4.4)	
Bachelor's degree or higher	17 (12)	7 (9.8)	10 (15)	
Unemployed	48 (34)	25 (35)	23 (33)	0.8
*Medical History*				
Diabetes mellitus	78 (56)	41 (58)	37 (54)	0.6
Hypertension	119 (85)	59 (83)	60 (87)	0.5
Hyperlipidemia	123 (88)	61 (86)	62 (90)	0.5
Heart failure	53 (38)	27 (38)	26 (38)	>0.9
Previous CAD	40 (29)	20 (28)	20 (29)	>0.9
Previous PCI	30 (21)	14 (20)	16 (23)	0.6
Previous CABG	1 (0.7)	1 (1.4)	0 (0)	>0.9
Current Smoking Status				0.4
Never smoker	79 (57)	41 (58)	38 (56)	
Current smoker	31 (22)	13 (18)	18 (26)	
Former smoker	29 (21)	17 (24)	12 (18)	
*Index ACS Event*				
Type of ACS				>0.9
STEMI	41 (29)	20 (28)	21 (30)	
NSTEMI	72 (51)	37 (52)	35 (51)	
Unstable angina	27 (19)	14 (20)	13 (19)	
Coronary anatomy				0.4
Single-vessel disease	50 (36)	23 (32)	27 (39)	
Multivessel disease	90 (64)	48 (68)	42 (61)	
Left ventricular ejection fraction, %	53 (43, 60)	53 (43, 59)	54 (42, 60)	>0.9
P2Y12 inhibitor at randomization				0.6
Prasugrel 10 mg	86 (61)	42 (59)	44 (64)	
Clopidogrel 75 mg	54 (39)	29 (41)	25 (36)	
*Baseline* Medications				
Beta-blocker	117 (84)	59 (83)	58 (84)	0.9
ACE inhibitor	33 (24)	18 (25)	15 (22)	0.6
ARB	85 (61)	41 (58)	44 (64)	0.5
SGLT2 inhibitor	69 (49)	35 (49)	34 (49)	>0.9
*Vital Signs and Laboratory Values*				
Systolic blood pressure, mmHg	119 (108, 134)	118 (108, 130)	119 (110, 134)	0.3
Diastolic blood pressure, mmHg	72 (65, 81)	68 (63, 79)	76 (69, 82)	0.006
Body mass index, kg/m²	29 (26, 33)	28 (25, 32)	29 (27, 34)	0.047
Creatinine, mg/dL	0.88 (0.72, 1.09)	0.85 (0.68, 1.06)	0.95 (0.73, 1.09)	0.2
eGFR, mL/min/1.73m²	94 (73, 106)	95 (66, 106)	89 (73, 105)	0.6
Hemoglobin A1c, %	6.6 (5.6, 9.3)	7.2 (5.7, 9.5)	6.3 (5.6, 8.4)	0.2

Data are presented as median (interquartile range) for continuous variables and n ( %) for categorical variables. P-values calculated using Wilcoxon rank-sum test for continuous variables and chi-square or Fisher's exact test for categorical variables.

Abbreviations: ACS, acute coronary syndrome; ACE, angiotensin-converting enzyme; ARB, angiotensin receptor blocker; CABG, coronary artery bypass grafting; eGFR, estimated glomerular filtration rate; IQR, interquartile range; NSTEMI, non-ST-elevation myocardial infarction; PCI, percutaneous coronary intervention; SGLT2, sodium-glucose cotransporter-2; STEMI, ST-elevation myocardial infarction.

The index ACS presentations included ST-elevation myocardial infarction (29 %), non-ST-elevation myocardial infarction (51 %), and unstable angina (19 %). Multi-vessel CAD was present in 64 % of participants. All participants were receiving evidence-based post-ACS therapies at baseline, including universal high-intensity statin and DAPT therapy, beta-blockers in 84 % of patients, and renin-angiotensin system inhibitors in 85 %. Additional lipid-lowering therapy included ezetimibe in 34 %. A total of 61 % were prescribed prasugrel and 39 % clopidogrel as their P2Y12 inhibitor.

At enrollment, participants demonstrated evidence of intensive lipid management during their index hospitalization and immediate post-discharge period, with a median baseline LDL-C of 57 mg/dL (IQR 44–73 mg/dL). Baseline platelet testing revealed a median platelet reactivity of 0 Ω (IQR 0–2 Ω), indicating adequate platelet inhibition in most participants. These baseline values were similar between treatment groups.

### 3.1 Treatment effects of the polypill vs usual care

In the polypill arm, there was a significant LDL-C reduction (-6.74 mg/dL, 95 % CI -12.03 to -1.46, *p* = 0.013) with no change in platelet reactivity (+0.55 Ω, 95 % CI -0.32 to 1.43, *p* = 0.21) from baseline to 30-day follow up (Table 2). In contrast, the usual care participants had no change in LDL-C (+0.53 mg/dL, 95 % CI -6.75 to 7.81, *p* = 0.89) and a significant worsening in platelet reactivity (+0.76 Ω, 95 % CI 0.09 to 1.44, *p* = 0.028). However, between-group differences were non-significant for LDL-C (LSM: 54.78 vs 59.36 mg/dL; difference -4.58 mg/dL, 95 % CI -12.68 to 3.52, *p* = 0.27) and platelet reactivity (2.17 vs 2.24 Ω; difference -0.08 Ω, 95 % CI -0.96 to 1.11, *p* = 0.88). The distributions of LDL-C and platelet reactivity at baseline and 30 days by treatment group are shown in **Supplemental Figure 1**.

**Table 2:** Effects of a polypill strategy after acute coronary syndrome versus usual care.

	Within Group Change from Baseline to 1 month				Between Group Difference	
	Polypill (*n* = 65)		Usual Care (*n* = 63)		Polypill vs Usual Care	
	Mean (95 % CI)	P-value	Mean (95 % CI)	P-value	LSM (95 % CI)	P-value
**Key Endpoints**						
LDL Cholesterol	-6.74	0.013	0.53	0.89	-4.58	0.27
(mg/dL)	(-12.03, -1.46)		(-6.75, 7.81)		(-12.68, 3.52)	
Platelet Reactivity	0.55	0.21	0.76	0.028	-0.08	0.88
(ohms)*	(-0.32, 1.43)		(0.09, 1.44)		(-0.96, 1.11)	
**Patient-Centered Endpoints**^						
Medication	0.61	0.002	0.21	0.14	0.13	0.47
Adherence (MMAS-8)	(0.24, 0.98)		(-0.07, 0.48)		(-0.23, 0.49)	
Treatment	1.81	0.47	0.65	0.79	-0.71	0.82
Satisfaction (TSQM)	(-3.17, 6.8)		(-4.26, 5.56)		(-6.81, 5.38)	
Quality of Life	8.18	<0.001	5.08	0.037	1.51	0.59
(SAQ)	(3.95, 12.4)		(0.32, 9.84)		(-4.06, 7.08)	
**Exploratory Subgroup with Poor Adherence at Baseline (MMAS< 6, *n*=36)**#						
LDL Cholesterol (mg/dL)	-11.36 (-18.45, -4.28)	0.003	-3.44 (-16.99, 10.11)	0.59	-9.32 (-20.62, 1.99)	0.10
Platelet Reactivity (ohms)	0.45 (-1.35, 2.26)	0.61	1.58 (-0.67, 3.84)	0.15	-0.74 (-3.23, 1.74)	0.55

Within-group changes analyzed using *t*-tests comparing follow-up values to baseline within each treatment arm. Between-group differences represent least squares means from linear regression models adjusting for baseline values, comparing polypill strategy versus usual care.

The polypill contained aspirin 81 mg, rosuvastatin 40 mg, and either clopidogrel 75 mg or prasugrel 10 mg administered once daily for 30 days.

⁎ Platelet reactivity was measured using whole blood impedance aggregometry with lower values indicating better platelet inhibition.

^ Medication adherence (MMAS-8) is scored 0–8 with higher scores indicating better adherence; scores <6 indicate low adherence, 6 to <8 medium adherence, and 8 high adherence. Treatment satisfaction (TSQM) and quality of life (SAQ) are scored 0–100 with higher scores indicating greater satisfaction and better quality of life, respectively.

# The subgroup analysis included participants with poor baseline medication adherence defined as MMAS-8 score <6. Abbreviations: LDL indicates low-density lipoprotein cholesterol; MMAS-8, 8-item Morisky Medication Adherence Scale; TSQM, Treatment Satisfaction Questionnaire for Medication; SAQ, Seattle Angina Questionnaire.

Across patient-reported outcomes, medication adherence increased significantly in the polypill group (mean change MMAS-8: +0.61, 95 % CI 0.24 to 0.98, *p* = 0.002) with a modest, non-significant improvement in usual care (mean change MMAS-8: +0.21, 95 % CI -0.07 to 0.48, *p* = 0.14). Quality of life improved significantly in both groups, with improvements in both the polypill arm (mean change SAQ Summary Score: +8.18, 95 % CI 3.95 to 12.4, *p* < 0.001) and usual care arm (mean change SAQ Summary Score: +5.08, 95 % CI 0.32 to 9.84, *p* = 0.037). Treatment satisfaction remained stable in both groups with no significant within-group changes. However, between-group differences were non-significant across all patient-reported measures (Table 2).

### 3.2 Treatment effects among participants with poor adherence at baseline

Among the 36 participants who demonstrated poor baseline medication adherence defined as MMAS-8 score < 6, there was a significant within-group LDL-C reduction in the polypill arm (-11.36 mg/dL, 95 % CI -18.45 to -4.28, *p* = 0.003) compared to no significant change in usual care (-3.44 mg/dL, 95 % CI -16.99 to 10.11, *p* = 0.59). The between-group difference approached statistical significance with a numerically larger LDL-C reduction favoring the polypill strategy (-9.32 mg/dL, 95 % CI -20.62 to 1.99, *p* = 0.10). For platelet reactivity, within-group changes were non-significant in both the polypill arm (+0.45 ohms, 95 % CI -1.35 to 2.26, *p* = 0.61) and usual care (+1.58 ohms, 95 % CI -0.67 to 3.84, *p* = 0.15), with no meaningful between-group difference (-0.74 ohms, 95 % CI -3.23 to 1.74, *p* = 0.55).

### 3.3 ED and hospitalization events over the follow-up period

Total ED (*n* = 13) and hospitalization (*n* = 10) events were comparable across both treatment groups throughout the follow-up period, with detailed reasons for ED visits and hospitalizations provided in Table 3. One participant in the usual care arm underwent non-elective percutaneous coronary intervention during a rehospitalization for ACS, and there were no cases of stent thrombosis during the follow-up period. Composite ED/hospitalization events were similar between groups (polypill vs usual care: 0.14 vs 0.18 events/participant, *p* = 0.67, Table 3).

**Table 3:** Thirty-day emergency department visits, all-cause hospitalizations, and composite events per participant by treatment arm.

Category	Overall (*n* = 140)	Polypill (*n* = 69)	Usual Care (*n* = 71)	P-Value
*Emergency Department Visits*				
Total ED visit events	13	6	7	0.86
*Primary reason for ED visit*				
Chest pain evaluation	7 (53.8 %)	3 (50.0 %)	4 (57.1 %)	
Cardiac-related, other	1 (7.7 %)	1 (16.7 %)	0 (0 %)	
Non-cardiac	5 (38.5 %)	2 (33.3 %)	3 (42.9 %)	
*Hospitalizations*				
Total hospitalization events	10	4	6	0.58
*Primary reason for hospitalization*				
ACS	1 (10.0 %)	0 (0 %)	1 (16.7 %)	
Heart failure	2 (20.0 %)	0 (0 %)	2 (33.3 %)	
Cardiac-related, other	4 (40.0 %)	3 (75.0 %)	1 (16.7 %)	
Non-cardiac	3 (30.0 %)	1 (25.0 %)	2 (33.3 %)	
*Composite Events*				
Total events	23	10	13	0.67
Composite events per participant	0.16	0.14	0.18	
*Safety Outcomes*				
Non-elective PCI performed	1 (0.7 %)	0 (0 %)	1 (1.4 %)	
Stent thrombosis	0 (0 %)	0 (0 %)	0 (0 %)	

Data shown as n ( %) unless otherwise specified.

Composite events per participant calculated as total events divided by number of participants in each group with P value derived from negative binomial regression model comparing event rates between groups.

ACS-related visits included evaluation for chest pain, dyspnea, or other symptoms concerning for recurrent acute coronary syndrome. Heart failure-related hospitalizations were for symptomatic volume overload. Cardiac-related other included arrhythmia evaluation and unspecified cardiac diagnosis.

Abbreviations: ACS, acute coronary syndrome; CI, confidence interval; ED, emergency department; PCI, percutaneous coronary intervention.

## 4 Discussion

This pilot randomized controlled trial demonstrates the feasibility and safety of implementing a polypill strategy that incorporates DAPT and statin therapy among socioeconomically disadvantaged patients in the immediate post-ACS period. Participants in the polypill arm demonstrated significant within-group improvements in LDL-C reduction and sustained antiplatelet activity at 30-day period with no safety concerns. Participants in the usual care arm had no change in LDL-C and a worsening in platelet reactivity. While between-group differences did not reach statistical significance, the observed trends toward enhanced benefits among patients with poor baseline adherence represent important mechanistic signals that may become more pronounced over longer follow-up as adherence patterns degrade, supporting the rationale for adequately powered, longer-duration trials.

The inclusion of P2Y12 inhibitors in our polypill formulation addresses a critical implementation gap in existing polypill literature and represents an important innovation for post-ACS care. While prior studies have demonstrated the efficacy of polypill-based strategies in primary prevention and chronic, stable secondary prevention settings, no randomized trial has evaluated a polypill strategy for managing patients following an ACS event in the subacute high-risk period immediately following index hospitalization [15,[22], [23], [24]]. The landmark SECURE trial demonstrated cardiovascular benefits with a polypill containing aspirin, ramipril, and atorvastatin among older patients with recent myocardial infarction, achieving a 24 % relative risk reduction in major adverse cardiovascular events [15]. However, this formulation did not include P2Y12 inhibitor therapy, which constitutes one of the most essential components of post-ACS management, particularly during the highest-risk period immediately following hospital discharge. The present study establishes that a polypill approach including P2Y12 inhibitors is both feasible and well-tolerated in a U.S. healthcare setting. Although theoretical concerns exist that polypill nonadherence could result in simultaneous discontinuation of all components, potentially causing greater harm than missing individual medications separately, our results demonstrated stable LDL-C and platelet inhibition in the polypill arm with comparable safety and satisfaction outcomes between groups. This represents an important step toward developing comprehensive polypill strategies that address the full spectrum of evidence-based therapies required for optimal post-ACS care in diverse healthcare environments and populations.

The modest improvements observed in this short-term pilot study must be interpreted within the context of baseline therapeutic saturation and medication adherence patterns following ACS. Importantly, all participants were prescribed evidence-based medications following their index hospitalization and randomized at a median of 15 days after percutaneous coronary intervention, representing an optimal baseline scenario with intensive therapy effects still apparent from the hospitalization period, potentially limiting the capacity to detect incremental benefit. Baseline lipid levels were excellent, with a median of 57 mg/dL, lower than the current treatment goal of LDL<70 mg/dL, and over one-third received ezetimibe add-on therapy. Similarly, participants demonstrated adequate platelet inhibition at baseline with a median platelet reactivity of 0 ohms, indicating optimal antiplatelet effects from prescribed dual antiplatelet therapy. The polypill arm maintained this adequate platelet inhibition without deterioration, while usual care demonstrated slight numerical worsening in platelet reactivity over the 30-day period, potentially signaling early adherence-related changes that may become more pronounced over time. The numerical improvements in lipid control observed with the polypill strategy, combined with maintained platelet inhibition, suggest potential clinical benefits which may be evident over longer-term follow-up. Medication adherence has been noted to deteriorate progressively over time following ACS events [25], and as adherence patterns degrade during transition from intensive hospital-based care to community management, the simplified medication regimen provided by the polypill approach may demonstrate increasingly apparent advantages [26]. Notably, the SECURE trial demonstrated no differences in LDL-C or blood pressure levels between polypill and usual care groups, yet achieved a 24 % relative risk reduction in major cardiovascular events, suggesting that biological surrogates may not fully capture polypill benefits in ACS [15]. A future trial extending follow-up for 6-months in the post-ACS period is underway (NCT07032389) to determine if a polypill-based approach may improve clinical outcomes in this vulnerable population.

A key strength of this study is the use of a pragmatic encapsulation-based polypill approach that addresses common implementation barriers associated with traditional polypill strategies. Rather than requiring chemical compounding or reformulation, our polypill contained individually FDA-approved medications that were over-encapsulated together, offering flexibility for dose adjustments while maintaining simplicity benefits, and this approach has been used in other polypill-based studies [22,27,28]. This approach provides robust personalization through multiple dose-titratable formulations without sacrificing adherence advantages. Each individual capsule has minimal cost, and the encapsulation process can be automated for larger-scale implementation. By eliminating the regulatory complexity and manufacturing costs associated with developing entirely new combination formulations, this pragmatic approach accelerates the pathway to clinical implementation while maintaining the core benefits of simplified medication regimens for improving adherence in high-risk post-ACS populations.

Additional strengths include its focus on a highly diverse (63 % Hispanic, 46 % Spanish-speaking participants), socioeconomically disadvantaged population that has been underrepresented in cardiovascular clinical trials, the availability of two polypills based on either prasugrel or clopidogrel prescription, and the use of objective biomarkers of medication adherence through platelet reactivity testing and lipid measurements. However, several limitations must be acknowledged. The 30-day follow-up duration captured early pharmacodynamic effects but may have been insufficient to capture the full clinical benefits of improved medication adherence, which typically manifest over longer time periods and multiple prescription and refill cycles. Enrollment at a median of 15 days after index ACS event occurred after the critical hospital-to-home transition period when medication reconciliation errors and adherence lapses are most common. In-hospital randomization with polypill initiation prior to discharge may have better captured the intervention's impact during this highest-risk window. The modest sample size limits the generalizability of findings and statistical power to detect clinically meaningful differences. The open-label design may have introduced bias in patient-reported outcomes, though objective biomarker endpoints mitigate this concern. Additionally, the study was not powered to evaluate differences in clinical cardiovascular events.

## 5 Conclusions

This pilot RCT demonstrates feasibility of a polypill strategy incorporating aspirin, P2Y12 inhibitors, and statins in the post-ACS period. While participants in the polypill arm demonstrated favorable reductions in LDL-C and improvements in adherence from baseline to follow up, the treatment effect of polypill vs. usual care on these outcomes was not significant. Adequately powered, longer-duration trials are needed to evaluate the potential clinical benefits of a polypill strategy in this high-risk post-ACS patient population.

## Role of funder

This study did not have any external funding and was funded internally with institutional research support to the Principal Investigator.

**Central Illustration**: POLY-ACS Trial

Caption: Study timeline from index ACS event, hospital discharge, outpatient baseline/randomization visit, and 30-day follow-up. After hospital discharge, participants were randomized to a once-daily polypill (aspirin 81 mg, rosuvastatin 40 mg, clopidogrel 75 mg or prasugrel 10 mg) versus usual care. Participants returned for repeat assessments at 30 days post-randomization. There was no significant difference in LDL-C or platelet reactivity at 30-days between groups. Abbreviations: ACS, acute coronary syndrome; PCI, percutaneous coronary intervention; IQR, interquartile range.

**Figure:**
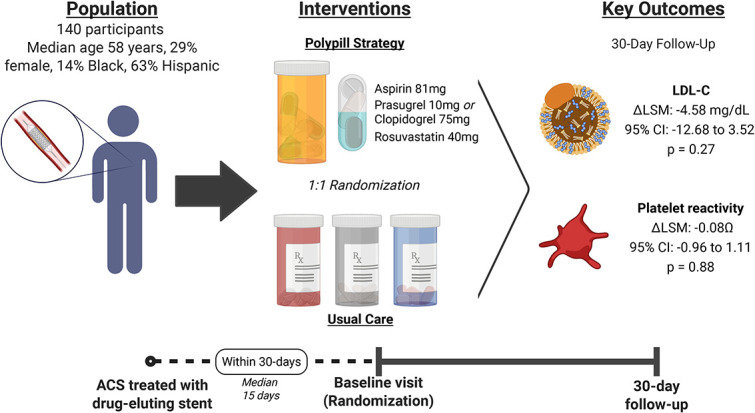

## Funding

This study did not have any external funding

## CRediT authorship contribution statement

**Juan David Coellar:** Writing – review & editing, Writing – original draft, Visualization, Validation, Supervision, Resources, Project administration, Methodology, Investigation, Data curation. **Neil Keshvani:** Writing – review & editing, Writing – original draft, Visualization, Validation, Supervision, Resources, Project administration, Methodology, Investigation, Funding acquisition, Formal analysis, Data curation, Conceptualization. **Anand K. Jain:** Validation, Software, Methodology, Formal analysis, Data curation. **Muhammad Shariq Usman:** Writing – review & editing, Writing – original draft, Visualization, Validation, Supervision. **Matthew W. Segar:** Writing – review & editing, Writing – original draft, Formal analysis, Data curation. **James W. Miller:** Writing – original draft, Resources, Methodology. **Myriam Bustillo-Rubio:** Writing – original draft, Validation, Project administration, Methodology, Investigation, Data curation. **Dharam J. Kumbhani:** Writing – review & editing, Writing – original draft, Validation, Project administration, Methodology, Investigation. **Mohanakrishnan Sathyamoorthy:** Writing – review & editing, Methodology, Investigation. **Javed Butler:** Writing – review & editing, Writing – original draft, Methodology, Investigation, Conceptualization. **Subhash Banerjee:** Writing – review & editing, Writing – original draft, Investigation. **Sean Yates:** Writing – review & editing, Methodology. **Thomas J. Wang:** Writing – review & editing, Writing – original draft, Resources, Methodology, Investigation, Conceptualization. **Ambarish Pandey:** Writing – review & editing, Writing – original draft, Visualization, Validation, Supervision, Resources, Project administration, Methodology, Investigation, Funding acquisition, Conceptualization.

## Declaration of competing interest

The authors declare the following financial interests/personal relationships which may be considered as potential competing interests:

Dr. Keshvani has received consultant fees from Heart Test Laboratories, Tricog Health, Idorsia Pharmaceuticals, and Science37. Dr. Segar reports non-financial support from Merck, honoraria outside the present study from Idorsia and Otsuka, is an advisor for AccurKardia, on the executive board for descendantsDNA, and the founder of ReCODE Medical. Dr Butler has been a consultant for Abbott, Adaptyx, American Regent, Amgen, AskBio, AstraZeneca, Bayer, Boehringer Ingelheim, Boston Scientific, Bristol Myers Squibb, Cardiac Dimension, Cardior, CSL Vifor, CVRx, Cytokinetics, Daxor, Diastol, Edwards, Element Sciences, Faraday, Idorsia, Impulse Dynamics, Imbria, Innolife, Intellia, Inventiva, Levator, Lexicon, Eli Lilly, Mankind, Medtronic, Merck, New Amsterdam, Novartis, Novo Nordisk, Pfizer, Pharmacosmos, Pharmain, Prolaio, Pulnovo, Regeneron, Renibus, Reprieve, Roche, Rycarma, Saillent, Salamandra, Salubris, SC Pharma, SQ Innovation, Secretome, Sequanna, Transmural, TekkunLev, Tenex, Tricog, Ultromic, Vera, and Zoll. Dr Banerjee is a board member of Elsevier and Cardiovascular Innovations foundation; and reports receiving institutional research grant from GE, Esperion, and Novartis; reports Honoraria from AngioSafe, Kaneka, and Terumo. Dr. Wang is named as a co-inventor on a provisional patent application related to machine technology for producing combination pills Dr. Pandey has received grant funding outside the present study from the National Institute of Health (R01Hl173838), American Heart Association, Applied Therapeutics, Roche, Ultromics, Astra Zeneca and Gilead Sciences and has received honoraria outside the present study as an advisor/consultant for Tricog Health Inc, Lilly, Rivus, Roche Diagnostics, Axon Therapies, Edward Lifesciences, Science37, Novo Nordisk, Bayer, Medical AI, Astra Zeneca, Baylor Scott and White Research Institute, iRhythm Technologies, Tenax, Boehringer Ingelheim, Tourmaline Bio, Merck, Sarfez Pharmaceuticals, Kardigan, Ultromics, Merck, Encarda, Kilele Health, Alnylam, Abbott, Anumana, Novartis, Antlia Biosciences, and Acorai. If there are other authors, they declare that they have no known competing financial interests or personal relationships that could have appeared to influence the work reported in this paper.

## Appendix Supplementary materials

Image, application 1

## References

[1] Tsao C.W., Aday A.W., Almarzooq Z.I., Alonso A., Beaton A.Z., Bittencourt M.S. (2022). Heart disease and stroke statistics-2022 update: a report from the American Heart Association. Circulation.

[2] Steen D.L., Khan I., Andrade K., Koumas A., Giugliano RP. (2022). Event rates and risk factors for recurrent cardiovascular events and mortality in a contemporary post acute coronary syndrome population representing 239 234 patients during 2005 to 2018 in the United States. J Am Heart Assoc.

[3] Rao S.V., O'Donoghue M.L., Ruel M., Rab T., Tamis-Holland J.E., Alexander J.H. (2025). 2025 ACC/AHA/ACEP/NAEMSP/SCAI guideline for the management of patients with acute coronary syndromes: a report of the American College of Cardiology/American Heart Association Joint Committee on clinical practice guidelines. Circulation.

[4] Thomas A., Gitto M., Shah S., Saito Y., Tirziu D., Chieffo A. (2023). Antiplatelet strategies following PCI: a review of trials informing current and future therapies. J Soc Cardiovasc Angiogr Interv.

[5] Ho P.M., Peterson E.D., Wang L., Magid D.J., Fihn S.D., Larsen G.C. (2008). Incidence of death and acute myocardial infarction associated with stopping clopidogrel after acute coronary syndrome. JAMA.

[6] Spertus J.A., Kettelkamp R., Vance C., Decker C., Jones P.G., Rumsfeld J.S. (2006). Prevalence, predictors, and outcomes of premature discontinuation of thienopyridine therapy after drug-eluting stent placement: results from the PREMIER registry. Circulation.

[7] Mathews R., Peterson E.D., Honeycutt E., Chin C.T., Effron M.B., Zettler M. (2015). Early medication nonadherence after acute myocardial infarction: insights into actionable opportunities from the treatment with adp receptor inhibitors: longitudinal assessment of treatment patterns and events after acute coronary Syndrome (TRANSLATE-ACS) study. Circ Cardiovasc Qual Outcomes.

[8] Colantonio L.D., Rosenson R.S., Deng L., Monda K.L., Dai Y., Farkouh M.E. (2019). Adherence to statin therapy among US adults between 2007 and 2014. J Am Heart Assoc.

[9] Crowley M.J., Zullig L.L., Shah B.R., Shaw R.J., Lindquist J.H., Peterson E.D. (2015). Medication non-adherence after myocardial infarction: an exploration of modifying factors. J Gen Intern Med.

[10] Dayoub E.J., Seigerman M., Tuteja S., Kobayashi T., Kolansky D.M., Giri J. (2018). Trends in platelet adenosine diphosphate P2Y12 receptor inhibitor use and adherence among antiplatelet-naive patients after percutaneous coronary intervention, 2008-2016. JAMA Intern Med.

[11] Mathews R., Wang W., Kaltenbach L.A., Thomas L., Shah R.U., Ali M. (2018). Hospital variation in adherence rates to secondary prevention medications and the implications on quality. Circulation.

[12] Nathan A.S., Geng Z., Eberly L.A., Eneanya N.D., Dayoub E.J., Khatana S.A.M. (2022). Identifying racial, ethnic, and socioeconomic inequities in the use of novel P2Y12 inhibitors after percutaneous coronary intervention. J Invasive Cardiol.

[13] Agarwal A., Mehta P.M., Jacobson T., Shah N.S., Ye J., Zhu J. (2024). Fixed-dose combination therapy for the prevention of atherosclerotic cardiovascular disease. Nat Med.

[14] Rao S., Jamal Siddiqi T., Khan M.S., Michos E.D., Navar A.M., Wang T.J. (2022). Association of polypill therapy with cardiovascular outcomes, mortality, and adherence: a systematic review and meta-analysis of randomized controlled trials. Prog Cardiovasc Dis.

[15] Castellano J.M., Pocock S.J., Bhatt D.L., Quesada A.J., Owen R., Fernandez-Ortiz A. (2022). Polypill strategy in secondary cardiovascular prevention. N Engl J Med.

[16] Friedewald W.T., Levy R.I., Fredrickson DS. (1972). Estimation of the concentration of low-density lipoprotein cholesterol in plasma, without use of the preparative ultracentrifuge. Clin Chem.

[17] Dyszkiewicz-Korpanty A., Olteanu H., Frenkel E.P., Sarode R. (2007). Clopidogrel anti-platelet effect: an evaluation by optical aggregometry, impedance aggregometry, and the platelet function analyzer (PFA-100). Platelets.

[18] Moon S.J., Lee W.Y., Hwang J.S., Hong Y.P., Morisky DE. (2017). Accuracy of a screening tool for medication adherence: a systematic review and meta-analysis of the Morisky Medication Adherence Scale-8. PLoS One.

[19] Atkinson M.J., Sinha A., Hass S.L., Colman S.S., Kumar R.N., Brod M. (2004). Validation of a general measure of treatment satisfaction, the treatment satisfaction questionnaire for medication (TSQM), using a national panel study of chronic disease. Health Qual Life Outcomes.

[20] Liberato A.C.S., São João T.M., Jannuzzi F.F., Landaas E.J., Wongchareon K., Rodrigues R.C.M. (2020). Treatment satisfaction questionnaire for medication (TSQM version 1.4): ceiling and floor effects, reliability, and known-group validity in Brazilian outpatients with hypertension. Value Health Reg Issues.

[21] Spertus J.A., Winder J.A., Dewhurst T.A., Deyo R.A., Prodzinski J., McDonell M. (1995). Development and evaluation of the Seattle angina questionnaire: a new functional status measure for coronary artery disease. J Am Coll Cardiol.

[22] Munoz D., Uzoije P., Reynolds C., Miller R., Walkley D., Pappalardo S. (2019). Polypill for cardiovascular disease prevention in an underserved population. N Engl J Med.

[23] Yusuf S., Joseph P., Dans A., Gao P., Teo K., Xavier D. (2021). Polypill with or without aspirin in persons without cardiovascular disease. N Engl J Med.

[24] Roshandel G., Khoshnia M., Poustchi H., Hemming K., Kamangar F., Gharavi A. (2019). Effectiveness of polypill for primary and secondary prevention of cardiovascular diseases (PolyIran): a pragmatic, cluster-randomised trial. Lancet.

[25] Colantonio L.D., Huang L., Monda K.L., Bittner V., Serban M.C., Taylor B. (2017). Adherence to high-intensity statins following a myocardial infarction hospitalization among Medicare beneficiaries. JAMA Cardiol.

[26] Rivera A., Campos B., Ceolin S., Godoi A., Castanha E., Campello Jorge C.A. (2023). Polypill-based strategy vs. usual care for secondary prevention of cardiovascular disease: a meta-analysis of randomized controlled trials. Eur J Prev Cardiol.

[27] Pandey A., Keshvani N., Wang TJ. (2022). Should polypills Be used for heart failure with reduced ejection fraction?. Circulation.

[28] Keshvani N, Rizvi SK, Khashami F, Jain A, Coellar JD, Bustillo-Rubio M, Rationale (2025). Design, and baseline characteristics of the polypill for heart failure with reduced ejection fraction (POLY-HF) trial. J Card Fail.

