# Supplementary material for: A polypill strategy for lipid lowering and anti-platelet therapy after acute coronary syndrome: A pilot randomized controlled trial
Source: Am J Prev Cardiol. 2026 Mar 7;27:101499. doi: 10.1016/j.ajpc.2026.101499 (PMC13261256; doi:10.1016/j.ajpc.2026.101499)
Supplement: Supplementary file 1 [file mmc1.docx]

**Supplemental Table 1:** Key inclusion and exclusion criteria

| **Inclusion** | **Exclusion** |
| --- | --- |
| - English- or Spanish-speaking patients with acute coronary syndrome undergoing percutaneous coronary intervention with drug-eluting stent placement - Prescribed aspirin, prasugrel/clopidogrel, and a statin at discharge - Randomization within 30 days of index hospitalization discharge. | - eGFR<30 ml/min/1.73m^2^ or end stage kidney disease - Need for inotropes or with cardiac index < 2.2 L/min/m^2^ - Receiving systemic anticoagulation (DOAC, Warfarin, etc.) - Contraindication, intolerance or allergic reactions to any of the polypill components (Aspirin, Prasugrel/Clopidogrel and Rosuvastatin) - Comorbidities that might be expected to limit lifespan within the 1-month study period - Inability to provide informed consent |
| Abbreviations: eGFR – estimated glomerular filtration rate; DOAC – direct oral anticoagulant | |

**Supplemental Figure 1**: Distribution of LDL cholesterol and platelet reactivity at baseline and 30 days by treatment group


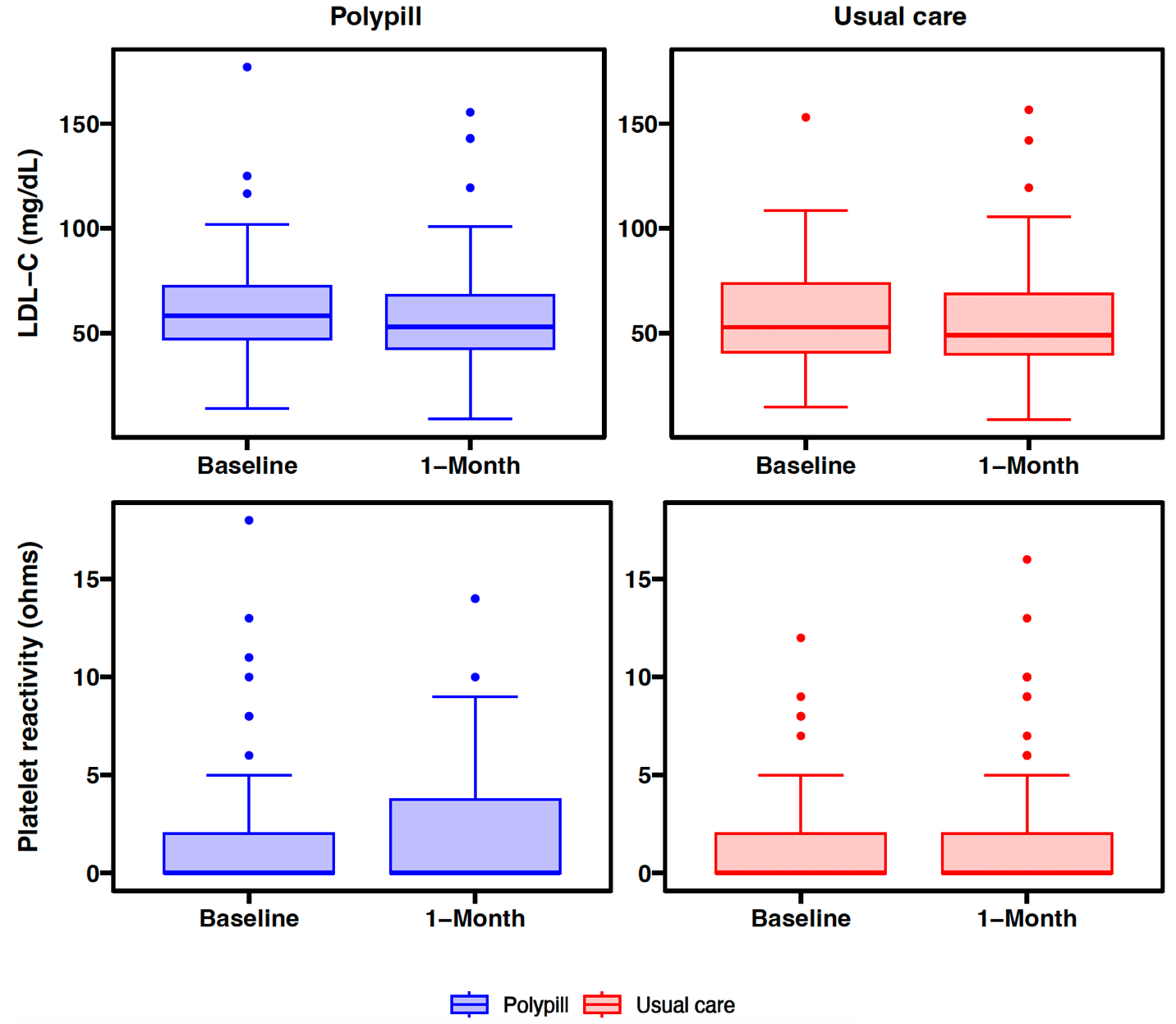
Caption: Box plots show the distribution of LDL cholesterol (top panels) and platelet reactivity measured by impedance aggregometry (bottom panels) at baseline and 1-month follow-up for polypill (left panels, blue) and usual care (right panels, red) groups. Boxes represent the interquartile range (25th to 75th percentile) with the median shown as the horizontal line within each box. Whiskers extend to 1.5 times the interquartile range, with individual outliers shown as dots.
